# Interference between Sentence Processing and Probabilistic Implicit Sequence Learning

**DOI:** 10.1371/journal.pone.0017577

**Published:** 2011-03-08

**Authors:** Dezso Nemeth, Karolina Janacsek, Gabor Csifcsak, Gabor Szvoboda, James H. Howard, Darlene V. Howard

**Affiliations:** 1 Institute of Psychology, University of Szeged, Szeged, Hungary; 2 Department of Psychology, The Catholic University of America, Washington, D. C., United States of America; 3 Department of Psychology, Georgetown University, Washington, D. C., United States of America; University of Maribor, Slovenia

## Abstract

**Background:**

During sentence processing we decode the sequential combination of words, phrases or sentences according to previously learned rules. The computational mechanisms and neural correlates of these rules are still much debated. Other key issue is whether sentence processing solely relies on language-specific mechanisms or is it also governed by domain-general principles.

**Methodology/Principal Findings:**

In the present study, we investigated the relationship between sentence processing and implicit sequence learning in a dual-task paradigm in which the primary task was a non-linguistic task (Alternating Serial Reaction Time Task for measuring probabilistic implicit sequence learning), while the secondary task were a sentence comprehension task relying on syntactic processing. We used two control conditions: a non-linguistic one (math condition) and a linguistic task (word processing task). Here we show that the sentence processing interfered with the probabilistic implicit sequence learning task, while the other two tasks did not produce a similar effect.

**Conclusions/Significance:**

Our findings suggest that operations during sentence processing utilize resources underlying non-domain-specific probabilistic procedural learning. Furthermore, it provides a bridge between two competitive frameworks of language processing. It appears that procedural and statistical models of language are not mutually exclusive, particularly for sentence processing. These results show that the implicit procedural system is engaged in sentence processing, but on a mechanism level, language might still be based on statistical computations.

## Introduction

Sentence processing works in a fast, automatic and unconscious way. It is widely accepted that during syntactic processing we decode the sequential and hierarchical combination of words, phrases or sentences according to previously learned and well-established rules. These rules, even if they exist, are represented in the mental grammar, the computational mechanisms and neural correlates of which are still much debated in the literature [1], [2]. Other key issue in neurolinguistic research is whether sentence processing solely relies on language-specific structures and mechanisms or is it also governed by domain-general computational principles [3], [4], [5]. We are trying to build a bridge between frameworks of sentence processing in order to find the ‘secret ingredient’ of this fundamental human skill.

There are two competing theoretical frameworks regarding the neural underpinnings of language capacities in the human brain. “Dual-system” theories attribute distinct, specialized and innate cognitive and neural components to the mental grammar and the mental lexicon respectively [6], [7], [8], [9], [10], [11]. According to one such dualistic model, the mental lexicon relies on the declarative memory system, while the mental grammar is subserved by structures involved in procedural memory [12]. The procedural memory system is responsible for gradual, implicit (non-conscious) learning and controlling motor and cognitive ‘skills’ and ‘habits’, especially those involving rules or sequences, such as riding a bicycle or using tools and other manipulated objects [13], [14], [15]. This system is rooted in frontal lobe/basal-ganglia circuits, in particular premotor regions, Broca's area and the caudate nucleus. It also encompasses other structures, including portions of superior temporal cortex and the cerebellum [12].

In contrast, “single-system” theories posit that words and grammatical rules are learned and utilized by a single non-language-specific system with a broad anatomical distribution [16], [17], [18], [19]. According to this view, grammatical rules are only descriptive entities; during actual language acquisition we learn the entire statistical structure of the language. Modern connectionist theories argue that learning, representation, and processing of grammatical rules and lexical items are the product of a network, which consists of a large number of simple inter-connected processing units, the connections of which are continuously adjusted on the basis of statistical contingencies in the environment [16], [18], [19]. In a recent paper, Conway and colleagues [20] provided evidence that speech perception is related to statistical learning. The statistical learning theory of language has been used to explain mechanisms of constructive grammar [21], language development [22], [23] and is also supported by studies of artificial language learning [24], [25], [26].

Thus, whereas dual-system theories link syntactic processing primarily to frontal brain regions and procedural memory, single-system theories suggest that grammar appears as the result of general statistical computations within a widespread neural network in the brain. Although implicit/procedural and statistical learning models offer apparently different interpretations for mental processes, a recent theoretical paper highlighted the similarities between the two principles and suggested that they are closely related [27]. The goal of the experiment reported here was to test whether sentence processing relies on general (non-linguistic) statistical computations supporting procedural learning.

During the past decade, new experimental paradigms emerged which successfully address both procedural and statistical motor learning. The Alternating Serial Reaction Time (ASRT) task [28], [29] was developed within the context of classical procedural-learning tests, the finger-tapping task and the Serial Reaction Time (SRT) task. Finger-tapping and SRT tasks involve both general skill and sequence-specific learning and they test basal ganglia and cerebellar functions [30], [31], [32]. The advantage of the ASRT task is that it enables separate parallel assessment of sequence-specific and general skill learning. In the classical SRT task, the structure of a sequence is deterministic with the stimuli following a simple cyclically repeating pattern (e.g. 213412134121341213412…, where numbers refer to distinct events within the repeating 21341 pattern). In contrast, repeating events alternate with random elements in the ASRT task. This means that the location of every second stimulus on the screen is determined randomly. If, for instance, the sequence is 1234, where the numbers represent locations on the screen, in the ASRT task the sequence of stimuli will be 1R2R3R4R, with ‘R’ representing a random element. Because fixed, sequence-specific and random stimuli are alternating, some sequences of three events (called ‘triplets’) occur more frequently than others. For example, in the above illustration 1×2, 2×3, 3×4 and 4×1 would occur often, whereas 1×3 or 4×2 would occur infrequently. Following previous studies, we refer to the former as *high-frequency* triplets and the latter as *low-frequency* triplets [29], [33]. In a typical ASRT task, participants are instructed to respond to different stimulus events (e.g., the same image appearing in one of four possible locations on the screen) by pressing different response keys (e.g., a separate letter on a keyboard assigned to each of the four image locations) as fast and accurately as they can. Earlier results have shown that as people practice the ASRT task, they respond more quickly to the high- than low-frequency triplets revealing probabilistic, sequence-specific learning [28], [29]. This learning is statistical in nature, because it depends on the frequency of the event sequences. In addition, the process is entirely implicit, as participants do not recognize the alternating structure of the sequences even after extended practice or when sensitive recognition tests are used to assess explicit knowledge [28], [29].

In the present study, we investigated the relationship between sentence processing and implicit sequence learning in a dual-task paradigm in which one task was a non-linguistic task (ASRT for measuring probabilistic implicit sequence learning), while others were a sentence comprehension task relying on syntactic processing and two control conditions. The majority of previous works on the relationship between language functions and the declarative/procedural system were based on manipulating regular and irregular forms of words [12]. Given that these tasks are not sensitive to other linguistic rules, such as word order, embedded structures etc., we used the comprehension of complex sentences, which we considered a more sensitive marker of grammatical processing. We selected two control conditions: a non-linguistic one (math condition) and a linguistic task (word processing task) where grammatical computations were not required, only the utilization of the mental lexicon. Since the ASRT task relies both on the procedural system and on statistical computations, we hypothesized that implicit sequence-specific learning would be attenuated by simultaneous sentence comprehension if the two tasks engage the same neurocognitive system(s). Finding interference would serve as direct evidence that operations for sentence processing depend on statistical computations of non-linguistic nature.

## Methods

### Ethics Statement

Ethics approval was obtained by Psychology Ethics Committee at University of Szeged, Institute of Psychology. All subjects provided signed informed consent agreements and received no financial compensation for their participation.

### Participants

Twenty-six students between 21 and 25 years (average age: 22.54, SD: 1.17; 4 male/22 female) from the University of Szeged participated in the study. Subjects did not suffer from any developmental, psychiatric or neurological disorders.

### Procedure

**Figure 1:**
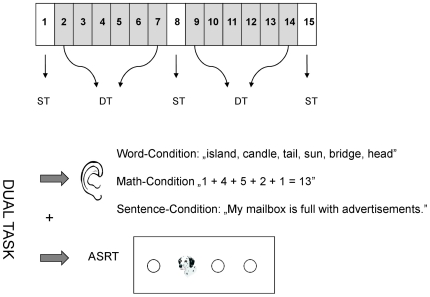
Schematic design of the experiment. The presentation order of the conditions was counterbalanced between subjects. In the ASRT task blocks 1, 8 and 15 were single task (ST) blocks without parallel task, whereas in other blocks (2–7; 9–14) our subjects had to perform one of the three parallel tasks as well (DT condition).

A dual-task paradigm [34], [35], [36], [37] was designed during which our subjects were instructed to perform the ASRT and a parallel task simultaneously (DT condition). Three types of parallel tasks were used: (1) sentence comprehension, (2) word recognition and (3) mathematical addition. Investigating the interference between sentence comprehension and procedural learning was the primary goal of the study, whereas the other two parallel tasks served as linguistic (word recognition) and non-linguistic (mathematical addition) control tasks. While both the sentence comprehension and word recognition tasks require access to the mental lexicon, mental grammar is only utilized by sentence comprehension. We used a within subject design with every subject performing all three parallel tasks, but with a different order. The subjects had a 5–10 minute-long rest between the different sessions. During these breaks, we collected demographic data (age, years of education, etc.). In order to objectively compare the degree of implicit learning in the three dual task sessions, we inserted three single task (ST) probe blocks (blocks 1, 8 and 15) during which the ASRT was the only task to perform (Figure 1).

### Tasks

#### Alternating Serial Reaction Time (ASRT) Task

We used a modification of the original ASRT task [33] in which a visual stimulus (a dog's head) appeared in one of the four empty circles on the screen and subjects had to press a key that corresponded to the actual spatial location (see Figure 1).

E-prime 1.2 was used for stimulus presentation and data collection. The computer was equipped with a special keyboard with four heightened keys (Y, C, B and M in the standard Hungarian IBM PC keyboard; the letter Y corresponds to the letter Z on standard English keyboards), each corresponding to one of the circles in left to right order. Before beginning the experiment, detailed instructions appeared on the screen. We emphasized that the aim was to try to respond as quickly and as correctly as possible.

The ASRT consisted of 15 blocks, with 85 key presses in each block: the first five stimuli were random events for practice purposes, after which an eight-element alternating sequence (e.g. 1R2R3R4R) repeated ten times. In order to objectively assess the degree of sequence-specific and general skill learning during the sessions, we inserted three probe blocks (blocks 1, 8 and 15), where no parallel task was present. Following Howard and Howard [28], stimuli were presented 120 ms after the previous motor response. As one block took about 1.5 minutes, each session with different DT conditions lasted approximately 30–35 minutes. Between stimulus blocks, the participants received feedback on the screen about their overall reaction time and accuracy. They could then rest for maximum 20 seconds before starting a new block.

For each subject, three different ASRT sequences (A: 1r3r2r4r; B: 4r3r1r2r; C:2r3r4r1r) were used for every session, and the occurrence of the different sequences was balanced across subjects and parallel tasks as well. Consequently, every sequence was used in all three DT conditions, but for different subjects.

To explore how much explicit knowledge subjects acquired about the sequence learning task, we administered a short questionnaire [29] after the experimental session. This questionnaire included specific questions such as “Have you noticed anything special regarding the task?” or “Have you noticed some regularity in the sequence of stimuli?”. The experimenter rated subjects' answers on a 5-item scale, where 1 corresponded to “Nothing noticed” and 5 to “Total awareness”. None of the subjects reported noticing the sequence in the ASRT task.

#### Parallel tasks

Every parallel task was presented in the auditory modality during the execution of the ASRT task in such a way that the parallel task items were read out loud by the experimenters and the subjects had to give a yes/no answer to each one. Participants were told to answer aloud as fast and accurately as possible after the actual task (word list, addition or sentence) was presented. The experimenter registered the answers and monitored continuously if participants followed the instructions.

Sentence processing (Sentence condition) - The subjects were instructed to listen to sentences and to decide after each one whether they were correct or not. Five to nine sentences were presented per ASRT block. Every sentence contained 6 words with half of the sentences being incorrect containing one of the following three error types: semantic, pragmatic or syntactic. Although we chose an error detection task to keep our subjects' attention focused on the task, the main emphasis was on overall sentence processing and not on error detection *per se*.

Word recognition (Word condition) - In the word processing condition the subject had to recognize words in lists containing 6 items. In order to control attention, the subjects had to decide if the list contained a non-word item that occurred at each position within a list with equal probability. Five to nine word lists were presented per ASRT block. Half of the lists contained a non-word, half of them did not.

Mathematical addition (Math condition) - The subject were presented with an addition of five items and the possible result (e.g. 4+9+2+1+3 = 19) after which they had to decide whether the result was correct or not. Similarly to the other two conditions, five to nine additions were presented per ASRT block. Half of the additions were correct, half were not.

Each list contained 6 items (words in the sentence comprehension or word recognition tasks and numbers in the math condition) in order to avoid varying working memory loads. In addition, we asked all subjects to name the most difficult parallel task at the end of the experiment.

### Statistical analysis

Both sequence-specific and general skill learning were evaluated by parameters obtained in the single task probe blocks of the ASRT. Sequence-specific learning was calculated by comparing RTs obtained for high- and low-frequency triplets, whereas general skill learning was determined by comparing RTs between the three probe blocks, regardless of triplet frequency. As expected [33], participants' accuracy was very high in the probe blocks (mean value >97% for all groups), so we focused on reaction time (RT) analysis. All significant results are reported together with the Greenhouse-Geisser ε correction factors, where applicable.

## Results

RT data were entered into a repeated-measures ANOVA, with TRIPLETS (high vs. low frequency), PROBE BLOCKS (blocks 1, 8 and 15) and CONDITIONS (sentence, word and math) as within-subject factors. The main question of the study was answered by the presence or absence of interaction between the CONDITION factor and one or both of general and sequence-specific skill learning.

**Figure 2:**
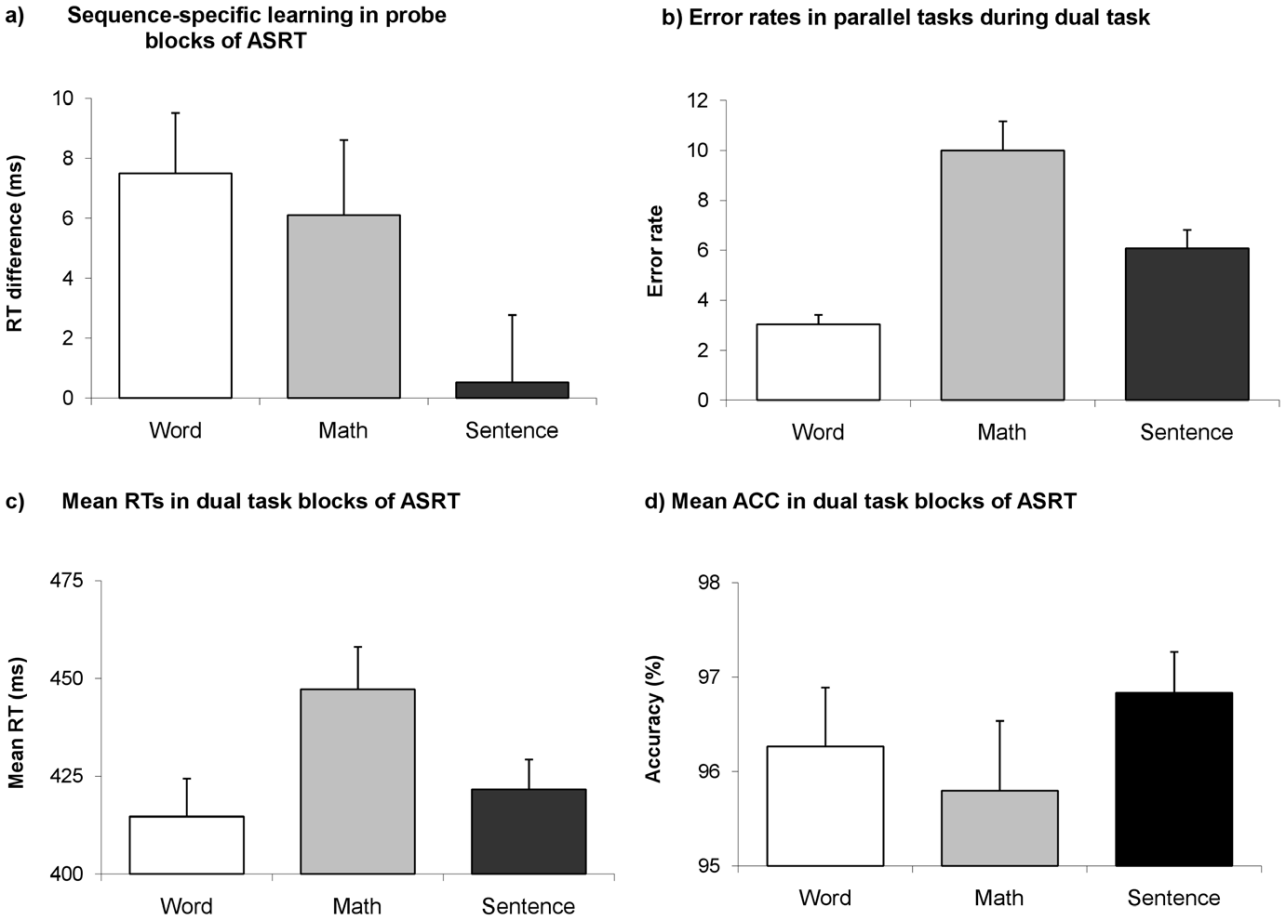
A) Mean RTs of sequence-specific learning (difference between high and low frequency triplets) in probe blocks of the ASRT task for all dual task conditions. There was significant sequence-specific learning in the Word and Math condition, but no learning in the Sentence condition. B) Error rates in parallel task during dual task. There were significantly more errors in the Math condition than in the other two conditions. C) Mean RTs in dual task blocks of the ASRT for all dual task conditions. The Math condition was the most difficult: the RTs differed significantly from the Word and Sentence conditions, while the latter two did not differ significantly from each other. D) Mean accuracy (ACC) in dual task blocks of the ASRT for all dual task conditions. The Math condition was the most difficult: participants were less accurate in the Math condition than in the Sentence condition, while the Word-Math and Word-Sentence conditions did not differ significantly from each other. Error bars indicate standard errors of the mean (SEM).

Repeated-measures ANOVA revealed sequence-specific learning (indicated by a significant main effect of TRIPLET: F(1,25) = 11.59, MSE = 224.21, p = 0.002, η_p_^2^ = 0.32), and general skill learning as well (indicated by a significant main effect of PROBE BLOCK: F(2,24) = 14.87, MSE = 639.95, p<0.001, η_p_^2^ = 0.55). The CONDITION×TRIPLET interaction was also significant (F(2,24) = 3.56, MSE = 190.01, p = 0.044, η_p_^2^ = 0.23), suggesting that sequence-specific learning differed between the three dual task conditions (see Figure 2A). General skill learning was not affected by the dual task conditions (CONDITION×BLOCK interaction: F(4,100) = 1.61, MSE = 1035.65, p = 0.18, η_p_^2^ = 0.06). Other interactions regarding the probe block data were not significant, nor was the main effect of CONDITION, suggesting that the overall RTs did not differ across conditions. Subsequent ANOVAs conducted separately for all dual task conditions revealed significant sequence-specific learning in both the word and math conditions (main effect of TRIPLET: F(1,25) = 13.85, MSE = 158.66, p = 0.001, η_p_^2^ = 0.36; F(1,25) = 5.86, MSE = 247.67, p = 0.02, η_p_^2^ = 0.19, respectively), whereas it was not significant in the sentence condition (F(1,25) = 0.06, MSE = 197.90, p = 0.82, η_p_^2^ = 0.002).

Error rates of the parallel task (Figure 2B) measured during the dual task conditions were significantly higher in the math condition (Mean: 10, SD = 5.95) than in the sentence comprehension (Mean = 6.08, SD = 3.84; p = 0.001) and word processing condition (Mean = 3.04, SD = 1.99; p<0.001), and word and sentence conditions differed from each other as well (p = 0.01). Mean overall RTs during the dual task blocks of the ASRT (Figure 2C) were significantly longer in the math condition (Mean = 447.22, SD = 55.54) than in the sentence comprehension (Mean = 421.71, SD = 39; p = 0.01) and word processing tasks (Mean = 414.71, SD = 49.41; p = 0.03), while we found no differences between the sentence and word conditions (p = 0.34). Moreover, mean accuracy during the dual task blocks of the ASRT (Figure 2D) was significantly lower in the math condition (Mean = 95.8, SD = 4.05) than in the sentence condition (Mean = 96.8, SD = 2.45; p = 0.04), while we found no differences between sentence-word (p = 0.35), and word-math conditions (p = 0.14). Finally, subjects' reports unanimously confirmed that the math condition was the most difficult. These results indicate that the modulation of sequence-specific learning was primarily affected by the nature of the parallel task and its underlying neural structures, and not by the difficulty of the parallel task itself.

## Discussion

In our study we found both general skill and sequence specific learning across tasks, however, we also found a clear dissociation - the sentence processing task diminished probabilistic implicit sequence learning, while the other two tasks did not produce a similar effect. This interference was not due to the complexity or relative difficulty of the parallel tasks, because (1) error rates of the parallel tasks were significantly higher in the math condition than for the linguistic tasks, (2) participants were significantly slower and less accurate in this math condition and (3) subjective reports confirmed that the math task was the most difficult.

The ASRT task is classically considered as an implicit motor learning task that depends on the procedural memory system [29]. The interference between the ASRT task and sentence processing but not word recognition partly supports the declarative/procedural model of language functions, according to which the mental grammar but not the lexicon engages the procedural system [12], [38]. The most important aspect of this study however, is that it goes beyond the classification of sentence processing as a procedural process. Sequence-specific learning in the ASRT task is based on unconscious detection of the conditional probabilities within the stimulus sequence as reflected in the high- and low-frequency triplets [39].

Several theories emphasized the highly probabilistic nature of language, which might indeed be linked to domain-general processes, such as statistical learning [5], [22], [26]. Artificial language learning is perhaps the most popular paradigm in this field, but to our knowledge, this is the first study demonstrating a link between language processing and a clearly non-linguistic probabilistic learning task (i.e. the ASRT task). Since the ASRT task shares features with both procedural and statistical learning, its interference with sentence processing might explain why syntactic processing has been previously associated with both types of learning. However more investigations with more language control conditions are needed to find out exactly which aspect of sentence processing interferes with probabilistic sequence learning.

Another interesting aspect of our results is that implicit sequence learning in the ASRT task is related to the motor system [30], [31], [32], which supports the motor theory of language [40] and might contribute to the evolutionary interpretations of language development [4].

In summary, we found that operations for sentence processing utilize resources underlying non-domain-specific probabilistic procedural learning in the human brain. Our study provides a bridge between two competitive frameworks of language processing. It appears that procedural/statistical models of language processing are not mutually exclusive, particularly for sentence processing. The implicit procedural system is crucial for sentence processing, but on a mechanism level, language might still be based on statistical computations.
